# Self-assembly of virus-like particles of porcine circovirus type 2 capsid protein expressed from *Escherichia coli*

**DOI:** 10.1186/1743-422X-7-166

**Published:** 2010-07-21

**Authors:** Shuanghui Yin, Shiqi Sun, Shunli Yang, Youjun Shang, Xuepeng Cai, Xiangtao Liu

**Affiliations:** 1 Lanzhou Veterinary Research Institute, Chinese Academy of Agricultural Sciences, State Key Laboratory of Veterinary Etiological Biology, Key Laboratory of Animal Virology of Ministry of Agriculture, Lanzhou 730046, China

## Abstract

**Background:**

Porcine circovirus 2 (PCV2) is a serious problem to the swine industry and can lead to significant negative impacts on profitability of pork production. Syndrome associated with PCV2 is known as porcine circovirus closely associated with post-weaning multisystemic wasting syndrome (PMWS). The capsid (Cap) protein of PCV2 is a major candidate antigen for development of recombinant vaccine and serological diagnostic method. The recombinant Cap protein has the ability to self-assemble into virus-like particles (VLPs) *in vitro*, it is particularly opportunity to develop the PV2 VLPs vaccine in *Escherichia coli*,(*E.coli *), because where the cost of the vaccine must be weighed against the value of the vaccinated pig, when it was to extend use the VLPs vaccine of PCV2.

**Results:**

In this report, a highly soluble Cap-tag protein expressed in *E.coli *was constructed with a p-SMK expression vector with a fusion tag of small ubiquitin-like modifiers (SUMO). The recombinant Cap was purified using Ni^2+ ^affinity resins, whereas the tag was used to remove the SUMO protease. Simultaneously, the whole native Cap protein was able to self-assemble into VLPs *in vitro *when viewed under an electron microscope. The Cap-like particles had a size and shape that resembled the authentic Cap. The result could also be applied in the large-scale production of VLPs of PCV2 and could be used as a diagnostic antigen or a potential VLP vaccine against PCV2 infection in pigs.

**Conclusion:**

we have, for the first time, utilized the SUMO fusion motif to successfully express the entire authentic Cap protein of PCV2 in *E. coli*. After the cleavage of the fusion motif, the nCap protein has the ability to self-assemble into VLPs, which can be used as as a potential vaccine to protect pigs from PCV2-infection.

## Background

Porcine circoviruses (PCVs), classified as a member of the family Circoviridae, are small icosahedral non-enveloped viruses (size ~17 nm) containing a circular single-stranded DNA molecule of about 1.7 kb. Two genotypes of PCV have been described. PCV1 was first isolated and characterized as a persistent contaminant of the PK-15 cell line and is considered as a non-pathogenic virus [1]. PCV2 is an etiologic agent that is associated with post-weaning multisystemic wasting syndrome (PMWS) [2,3]. PMWS is currently considered to be an important infectious swine viral disease that has a serious economic impact on the pig farming industry. Clinically, pigs affected with PMWS, frequently at 5 to 18 weeks of age, are characterized by pallor, progressive weight loss, fever, difficulty in breathing, enlarged lymph nodes, and, occasionally, diarrhea and jaundice. The morbidity rate is usually low, but case fatality can be more than 50% in affected herds [4].

The PCV2 genome contains two open reading frames (ORFs). ORF1 encodes the replicase (Rep and Rep') proteins located on the viral plus strands, which initiates viral replication. ORF2 encodes the major structural capsid (Cap) protein, which is a sole structural protein of the viral coat [5] and also the major immunogenic protein that works together with the principal carrier of type-specific epitopes [6]. The Cap protein is highly immunogenic and reacts strongly with the serum of PCV2-infected pigs. Therefore, it is a good candidate antigen for the design of new recombinant vaccines against PCV2 infection and for the development of serologic tests. There are several PCV2 vaccines currently on the market. The CIRCOVAC^® ^(Merial Com, lyon, France), which is an inactivated PCV2 vaccine. The vaccine of Suvaxyn PCV2 One Dose (Fort Dodge Animal Health, Fort Dodge, IA) was the first PCV2 vaccine approved for commercial use in the United States by the United States Department of Agticulture. This vaccine is a chimeric virus that was developed to have the PCV2 capsid and the PCV1genome. Another available two vaccines are subunit vaccine, Ingelvac^® ^CIRCOFLX™ (Boehringer Ingelheim Vermdedica Inc, St. joseph, MO) and Circumvent PCV (Intervet Inc, Millsboro, DE), which are the capsid-based subunit vaccine expressed in inactivated baculovirus. In recent years, two main expression systems, including prokaryocytes and eukaryocytes, have been applied to express Cap protein as an antigen for animal immunization against PCV2. The prominent characteristic of the recombinant Cap protein is its ability to independently self-assemble to form virus-like particles (VLPs) in eukaryocytes, such as insect-baculovirus and yeast expression systems [5,6]. However, few papers have reported on the production of successful Cap protein VLPs in *Escherichia coli *(*E. coli*) [7].

This is the first report on the production of Cap protein VLPs of PCV2 in *E. coli*. The small ubiquitin-like modifier (SUMO) fusion expression system is used to successfully express the whole native Cap (nCap) protein by making it highly soluble in *E. coli*. The SUMO tag is subjected to cleavage with SUMO protease. Simultaneously, the whole nCap protein self-assembles into VLPs *in vitro*.

## Materials and methods

### Construction of expression vectors with the fusion tag of SUMO

**Figure 1:**
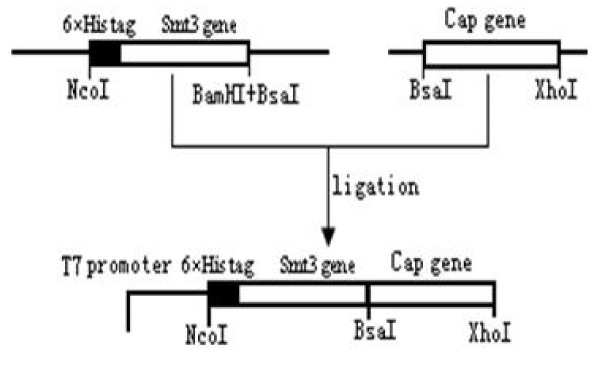
**Scheme for the construction of the expression vector p-SMK-Cap with SUMO motif**.

The SUMO (Smt3) gene was amplified from *Saccharomyces cerevisiae *with primers Smt3F (5'-GCCATGG (*Nco*I) GTCATCACCATCATCATCAC (6 × His) GGGTCGGACTCAGAAGTCAATCAA-3') and Smt3R (5'-GGATCC (*Bam*HI) GAGACC (*Bsa*I) TTAAGGTCTC (*Bsa*I) AACCTCCAATCTGTTCGCGGTG-3'). A *Nco*I restriction site followed by a 6 × His code sequence was incorporated into the 5' end of smt3. A 23-nucleotide sequence containing *Bsa*I on both positive and negative strands and a *Bam*HI restriction site were added to the 3' end of smt3 gene. After double digestion with restriction enzymes *Nco*I and *Bam*HI, the amplified SUMO gene was inserted into a pET-28a vector (Novagen) and digested with the same enzymes. The resultant plasmid was designated as pSMK (Fig. 1).

### Cloning the Cap gene and construction of a recombinant expression vector

A pair of expression primers of ORF2 was designed with the PCV2 sequences in the Genbank database (accession numbers FJ948168 and FJ948167). The full-length Cap gene (702 bp, the genotype is PCV2b) of PCV2 was amplified, using PCR from recombinant complete PCV2 genome plasmid previously stored in our laboratory, with the following primers: the upstream primer (CGTGGTCTCCAGGTATGACGTATCCAAGGAGGCGT) containing the *Bsa*I site and the downstream primer (TATCTCGAGTCAAGGGTTAAGTGGGGGGTCTTT) containing the *Xho*I site. The PCR product was purified and then digested with *Bsa*I and *Xho*I (NEB), cloned into p-SMK, and screened by transformants using restriction enzyme analysis. The recombinant plasmid was designated as p-SMK-Cap, which was transformed into the host, BL21-codon-Plus (DE3)-RIL strain (Stratagene).

### Expression and purification of recombinant Cap protein

An overnight culture of *E. coli *cells in Luria-Bertani (LB) medium (plus 34 μg/ml chloramphenicol and 80 μg/ml kanamycin) containing recombinant p-SMK-Cap plasmid was diluted 1:50 in 0.2 L fresh LB medium and incubated at 37°C until cells reached mid-log growth. The remainder of the culture was induced by the addition of isopropyl-β-thiogalactopyranoside (IPTG) to a final concentration of 0.1 mM and incubated further for 20 h at 20°C with shaking at 180 rpm/min.

Cells were harvested by centrifugation at 5000 g for 15 min at 4°C. The cell pellet was resuspended in 50 ml of 50 mM Tris-HCl buffer (1.0% Triton X-100, pH 8.0) by stirring in an ice-cold water bath. The suspension was disrupted on ice using ultrasonic cell crusher and was centrifuged at 12000 g for 30 min at 4°C, and collected using a 0.45 μm filter membrane, then equilibrated with three columns of Tris-HCl buffer (20 mM imidazole, 150 mM NaCl, pH 8.0) in a 70 ml column. The supernatant was transferred to the column and incubated for 30 min at 4°C. Superfluous recombinant proteins were washed with a Tris-HCl buffer (30 mM imidazole, pH 8.0) until the protein indicator cannot turn blue in a Bradford reagent (v/v: 5% of 95% ethanol, 10% of 88% phosphoric acid, and 0.07 mg/ml Coomassie brilliant blue G-250). Bound protein was eluted with a Tris-HCl buffer (300 mM imidazole, 150 mM NaCl, pH 8.0) until the protein indicator cannot turn blue. The eluted production was concentrated at 4°C. Then, 2 ml SUMO protease buffer (50 mM Tris-HCl, 0.2% Igepal, 1 mM DTT, pH 8.0) was added into the solution. The quantity of the purified Cap-tag protein was obtained by the Bradford assay (Sigma-Aldrich, USA) using bovine serum albumin as a standard. It was analyzed through 15% SDS-PAGE and stored at 4°C for further assay.

### Cleavage of a SUMO tag from a Cap-tag protein to yield authentic VLPs of Cap

A cleavage reaction assay was performed containing 1 × SUMO protease buffer, 40 μl SUMO protease (1 unit/μl, Invitrogen), 40 μg fusion protein, and water added to a total volume of 400 μl, and incubated for 5 h at 16°C.

The cleavage reaction was diluted with 2 ml binding buffer (50 mM Tris-HCl, 150 mM NaCl, pH 8.0), mixed with 200 μl resins, and bound for 50 min using gentle agitation to keep the resin suspended. The supernatant was transferred into an ultrafiltration tube and processed at 4°C and 2500 g to a final volume of 500 μl. The purified tagless nCap protein was identified through 15% SDS-PAGE. The VLP preparations were dialyzed against phosphate-buffered saline (PBS, pH 7.4) to a final volume of 30 μl and further confirmed by electron microscopy.

### Western blotting

Cap-tag and nCap, 30 μg respectively, which were transferred to an Immobilon-P transfer membrane (Millipore Corporation, Bedford, MA, USA) in a transfer buffer (20 mM Tris-HCl, 190 mM glycine, 0.1% SDS, 20% methanol, pH 8.3) using a Mini-protean^® ^tetra cell (Bio-Rad, USA) at 0.8 mA/cm^2 ^for 2 h. The membranes were blocked with 5% skim milk powder in PBST (PBS containing 0.05% Tween 20) and incubated with anti-His monoclonal antibody (mouse, 1:5000), (Sigma, USA) followed by a peroxidase-conjugated anti-mouse antibody. The DAB reagent was used to develop the signal

### Transmission electron microscopy

For TEM studies, 25 μg of the nCap VLPs were adsorbed onto a copper grid (200 mesh) for 2.5 min at room temperature. Then, the grids were dried gently using filter paper. After staining with 3% phosphotungstic acid (PTA) for 2.5 min, the excess liquid was removed, and the samples were viewed using a TEM (Hitachi, H-7100FA) at an acceleration voltage of 75 kV.

The nCap VLPs were observed by immunoelectron microscopy. Anti-PCV2 serum (1:100) from 60 day-old PCV2-vaccinated pig, was reacted with nCap VLPs at 4°C overnight. The products were centrifuged at 3500 g for 10 min. The pellet was dissolved in PBS. The prepared grid was stained by PTA and visualized by TEM.

## Results

### Expression, purification, and characterization of Cap protein

**Figure 2:**
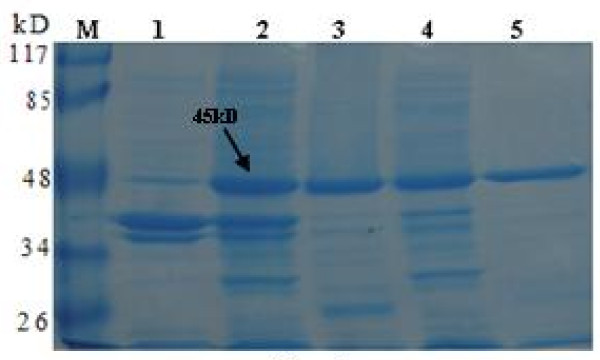
**SDS-PAGE analysis of the Cap-tag protein expression in *E. coli***. Lane M, molecular weight marker; lane 1, bacterial lysates from cells without IPTG; lane 2, bacterial lysates from IPTG-induced cells in *E. coli*; lane 3, pellet from bacterial lysates; lane 4, supernatant of bacterial lysates; lane 5, purified recombinant Cap-tag protein.

**Figure 3:**
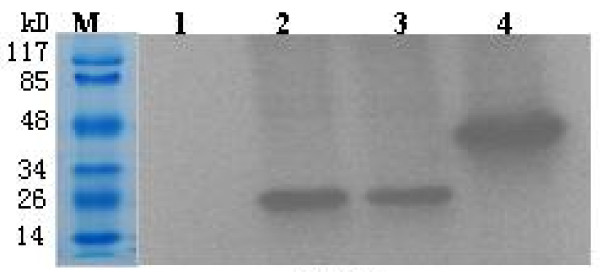
**Western blotting analysis of different Cap proteins with anti-PCV2 pig sera**. M, molecular weigh standard; Lane 1, uninduced recombinant *E. coli*; lanes 2 and 3, nCap protein cleavage of SUMO + 6 × His tag; lane 4, recombinant fusion Cap protein with SUMO + 6 × His tag.

The whole Cap gene was amplified by PCR of PCV2 genomic DNA and cloned into p-SMK expressed vector with the SUMO tag (5'-6 × His-SUMO-Cap-3') (Fig. 1). The recombinant strains were induced at 20°C by IPTG. The products were analyzed by SDS-PAGE. The Cap-tag in *E. coli *is highly water-soluble. The supernatant of cell lysis was purified on Ni^2+ ^affinity resins that have the ability to specifically bind to His_6_-tag polypeptide. The purity of the Cap-tag protein is greater than 90%, so it may be used as a template in the next protease cleavage reaction (Fig. 2). The reaction of Cap-tag and nCap may with anti-PCV2 pig sera and was observed by Western blotting (Fig. 3).

### Cleavage of His-Smt3 tag and assembly of Cap protein VLPs

After the cleavage of the His-Smt3 tag with SUMO protease from the Cap-tag, the mixture was again passed through Ni^2+ ^affinity resins. The cleaved His-Smt3 tag and the SUMO protease bind to the column, whereas nCap protein (about 26kDa) can be recovered in the flow-through and observed by SDS-PAGE.

### Characterization of PCV2 nCap protein VLPs from *E. coli*

**Figure 4:**
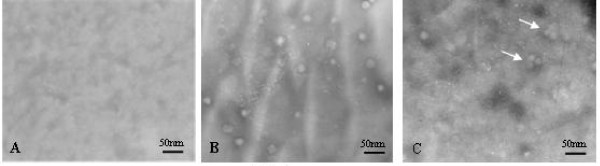
**TEM images of recombinant Cap protein VPLs of PCV2**. The recombinant Cap-tag protein (A) and Cap protein VPLs after cleavage of the SUMO tag (B); immunoelectron microscopy image of the Cap protein VPLs (C). Scale bar is 50 nm.

TEM and immunoelectron microscopy of the nCap protein show that it can assemble into Cap-like particles with diameter ranging from 15 to 20 nm (Fig. 4A and 4B), and an anti-PCV2 antibody that reacts with Cap VLPs antigen to produce complexs(Fig. 4C). The results suggest that the Cap protein expressed from *E. coli *assembles into VLPs.

## Discussion

It is usually difficult to express the whole Cap protein in *E. coli *possibly because of the specific amino acid at the N terminus of a nuclear localization signal (NLS) [8]. In whole Cap protein expression process, additional fusion protein partners, such as glutathione *S*-transferase or maltose binding protein, are able to achieve expression in minute amounts [9,10]. Only the deletion of the whole NLS domain may allow for the expression of a large amount of the Cap protein. However, the NLS plays an important role forming the self assembly of Cap-like particles and retains the antigenic function of the protein [11]. The N terminus of the Cap protein of the NLS domain is rich in arginine residues, which is a low-usage codon in *E. coli. *Thus, by using codon optimization to achieve Cap protein [12] and to overcome the difficulty of expressing an authentic Cap protein and proceeding to form VLPs *in vitro*, we recently constructed a SUMO fusion protein expression system to produce the high-level water-soluble Cap protein of PCV2 in *E. coli*.

Each prokaryocyte and eukaryocyte expression system has advantages and limitations. In contrast, *E. coli *has been a successful host for high expression levels of many heterologous proteins because of its relative simplicity, low cost, efficient generation time, and fast high-density cultivation. Nevertheless, challenges need to be overcome, such as proteolytic degradation of the target protein, misfolding, formation of inclusion bodies, and successful expression of soluble heterologous proteins. Some fusion motifs or partners are frequently employed to resolve the problems during the construction of expression vectors in *E. coli *[13-15].

The SUMO fusion expression system offers advantages over other fusion technologies, including improved soluble expression, proper folding, protection from degradation, and simplified purification and detection [16]. To differentiate from other proteases that recognize a peptide sequence and after cleaving, the fusion tag can generate extraneous amino acids at the N terminus of the target protein [17]. The SUMO protease recognizes the tertiary structure of the SUMO tag, is accurate and efficient for the generation of native N-terminal amino acids, and does not result in extraneous residues at the N terminus of the target protein in recombinant proteins [16]. These demonstrate its authentic traits.

VLPs mimic the structure of authentic virus particles and present viral antigens in a more authentic conformation and biological function. Therefore, they are easily recognized by the immune system and able to stimulate both B-cell and T-cell immune responses [18-20]. In particular, many VLPs are non-infectious because, although they completely lack the viral DNA or RNA genome, they are safer than attenuated or chemically inactivated live viruses. This striking feature of VLPs will likely contribute to the effectiveness of its use in vaccination as a strategy for controlling diseases [21-24].

Results of this research indicate that the VLPs of Cap protein of PCV2 is successfully expressed in *E. coli *as highly soluble substances. The study also shows that the fusion tag is subjected to cleavage with the SUMO protease. Simultaneously, the whole Cap protein is able to self-assemble into VLPs in vitro. TEM confirmed that its shape and size are similar to Cap isolated from the cell culture. The antigenic property of Cap protein VLPs was confirmed by Western blotting and immunoelectron microscopy.

## Conclusions

In summary, we have, for the first time, utilized the SUMO fusion motif to successfully express the entire authentic Cap protein of PCV2 in *E. coli*. After the cleavage of the fusion motif, the nCap protein has the ability to self-assemble into VLPs, which can be used as a potential VLPs vaccine to protect pigs from PCV2-infection.

## Abbreviations

PCV2: porcine circovirus type 2; nCap: native capside; PMWS: post-weaning multisystemic wasting syndrome; VLPs:virus-like particles; SUMO: small ubiquitin-like modifier; PCR: polymerase chain reaction; DNA: Deoxyribonucleic Acid; ORF: Open Reading Frame; TEM: transmission electron microscopy; PTA: phosphtungstic acid.

## Competing interests

The authors declare that they have no competing interests.

## Authors' contributions

SY focused on expression and purification of Cap protein, did do SDS-PAGE, Western blotting and drafted the manuscript. SS conducted recombinant expression vector with SUMO tag. SYg and YS contributed to the interpretation of the findings and revised the manuscript, carried out TEM. XC and XL edited the manuscript. All authors read and approved the final manuscript.
